# Targeting Skin Cancer with Natural Bioactive Compounds: From Molecular Mechanisms to Application Strategies

**DOI:** 10.3390/ph19060919

**Published:** 2026-06-11

**Authors:** Yuan Gao, Zesen Fang, Yan Xu, Yuyang Guo, Silin Liu, Haonan Dong, Jianghan Luo, Lijun Yan

**Affiliations:** School of Pharmacy, Engineering Research Center of Natural Antineoplastic Drugs, Ministry of Education, Harbin University of Commerce, Harbin 150076, China; fangzeseny024@s.hrbcu.edu.cn (Z.F.); xuyany023@s.hrbcu.edu.cn (Y.X.); guoyuyangy023@s.hrbcu.edu.cn (Y.G.); liusilin@s.hrbcu.edu.cn (S.L.); donghaonany025@s.hrbcu.edu.cn (H.D.); 102690@hrbcu.edu.cn (J.L.); ylj@hrbcu.edu.cn (L.Y.)

**Keywords:** skin cancer, natural products, apoptosis, photoprotection, antioxidant

## Abstract

Skin cancer presents a significant global health burden with rising incidence. The side effects of current therapies and the emergence of drug resistance necessitate the exploration of alternative and complementary strategies. Natural products, with their long history of use in treating skin disorders, have emerged as a promising source of novel therapeutic agents. This review comprehensively elucidates the potential efficacy of natural bioactive compounds in both preventing and treating skin cancer. We summarize the molecular mechanisms through which key natural bioactive compounds exert their anti-skin cancer effects, including induction of apoptosis, inhibition of proliferation and metastasis, anti-inflammatory and antioxidant activities, DNA damage repair, and photoprotection. Furthermore, we discuss the biological barriers relevant to skin cancer therapy using natural bioactive compounds and link them to corresponding delivery strategies, while identifying key translational challenges. In conclusion, natural bioactive compounds offer a multi-targeted and synergistic approach against skin carcinogenesis, holding substantial promise as sources of adjuvant therapies and chemopreventive agents to improve patient outcomes.

## 1. Introduction

Cutaneous malignancies, including basal cell carcinoma (BCC), squamous cell carcinoma (SCC), and melanoma, are increasing worldwide and are closely associated with ultraviolet exposure, genetic predisposition, and immune status [1]. Although modern medicine has made significant progress in the diagnosis and treatment of skin cancer (e.g., surgical excision, radiotherapy, chemotherapy, targeted therapy, immunotherapy), it includes problems such as the poor response of some patients to existing therapies, treatment-related toxicities, and poor prognosis for patients with advanced or metastatic disease [2]. Although many skin cancers are more amenable to prevention and early detection than several internal malignancies through regular skin examinations, surveillance of high-risk individuals, and timely management of precancerous lesions, the continuous increase in malignant and metastatic cases still highlights the urgent need for safer, effective, and complementary therapeutic strategies. Therefore, natural bioactive compounds with multi-targeted anticancer, photoprotective, antioxidant, and anti-inflammatory properties remain highly relevant for skin cancer prevention and treatment.

The use of natural products in the treatment of skin diseases has been practiced for thousands of years. Traditional Chinese medicine (TCM) and its active ingredients are also major sources of natural products. In modern research, there is also considerable investigation into the use of medicinal plants and their active compounds for the treatment of skin cancer. Several representative studies illustrate this potential. *Astragalus membranaceus* has been reported to protect against UVB-induced skin inflammation and photoaging by inhibiting nuclear factor kappa-B (NF-κB) activity and matrix metalloproteinase-1 (MMP-1) expression in Hs68 human dermal fibroblasts [3]. *Panax ginseng* inhibited chemically induced skin cancer in Swiss albino mice by increasing antioxidant enzyme activities, including superoxide dismutase (SOD) and catalase (CAT), elevating glutathione (GSH) levels, and reducing lipid peroxidation [4]. *Phellinus linteus* inhibited melanoma growth by inducing S-phase arrest and apoptosis in A375 cells [5]. Together, these findings suggest that medicinal plants and their active compounds may provide useful sources for skin cancer prevention and treatment research.

**Figure 1:**
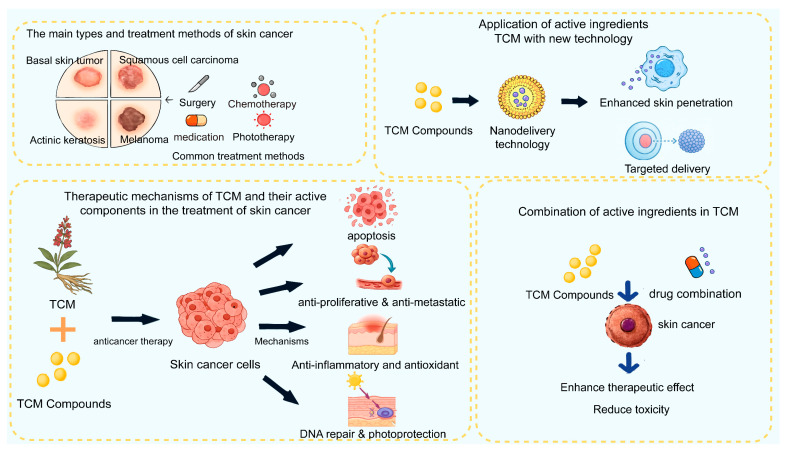
The primary applications of natural products in the treatment of skin cancer.

This review summarizes the potential applications of TCM-derived bioactive compounds in skin cancer prevention and treatment, with particular attention to their integration with emerging technologies (Figure 1).

## 2. Main Types and Pathogenesis of Skin Cancer

**Figure 2:**
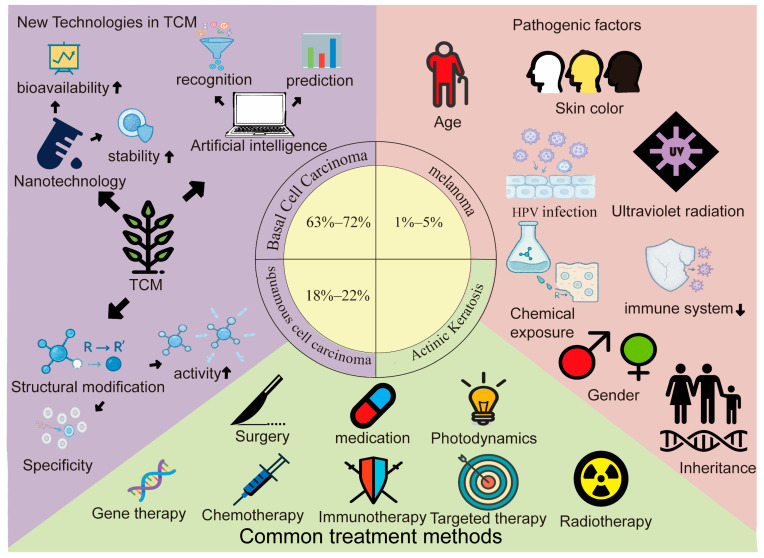
The main types and pathogenesis of skin cancer. The arrows in the left panel indicate the relationships between TCM-related new technologies and their corresponding improvements; different background colors distinguish new technologies in TCM, pathogenic factors, and common treatment methods.

Skin cancer is one of the most common cancers worldwide, with continuously increasing incidence and mortality. Its development is associated with multiple factors, including ultraviolet exposure, age, skin type, sex, genetic susceptibility, immune status, and preventive behaviors. There are two types of skin cancer, the rarer but more serious melanoma and non-melanoma skin cancers, with non-melanoma skin cancers including basal cutaneous tumors (BCC), which account for 70% of non-melanoma skin cancers, and squamous cell carcinomas (SCC), which account for 25% of non-melanoma skin cancers (NMSC), and other rarer types [6], as shown in Figure 2.

BCC is a malignant tumor originating from the basal cell layer of the skin that is usually slow-growing and locally invasive but rarely develops distant metastases [7]. SCC is a malignant neoplasm of cutaneous or mucosal squamous epithelium with characteristic squamous differentiation. It arises from gene mutations driven by multiple etiologic factors, such as chronic ultraviolet exposure, chemical carcinogens, human papillomavirus (HPV) infection, immune suppression, and genetic susceptibility [8]. Additionally, actinic keratosis (AK) is generally regarded as a precancerous lesion, and a certain proportion of untreated AK will progress to skin cancer. Chronic UV radiation is the main cause of AK [9].

### 2.1. Treatment of Non-Melanoma Tumors

The diagnosis of non-melanoma is mostly made through symptom evaluation and pathological examination, and the individual treatment method is determined by the tumor location, thickness, skin coverage and type, of which surgical resection is the main treatment means, and the other treatment methods include non-surgical therapy with topical drug method, photodynamic therapy, destructive therapy, targeted therapy and immunotherapy mainly for advanced and metastatic non-melanoma. Radiotherapy is mainly used in selected patients, such as those who are not suitable surgical candidates or those with specific high-risk or advanced lesions. Combination therapy has also emerged as an important strategy to improve local control and therapeutic outcomes [10]. Among these therapies, radiotherapy may be associated with local tissue damage and treatment-related adverse effects; non-surgical therapies require strict indications, and targeted therapies may be limited by drug resistance and insufficient tumor specificity [11].

### 2.2. Therapeutic Options for Melanoma

Melanoma is a highly malignant skin tumor that is mainly associated with ultraviolet radiation, genetic factors, malignant transformations in moles, dysfunctions of the immune system, and exposure to certain chemicals. The genes and signaling pathways include B-Raf proto-oncogene, serine/threonine kinase (BRAF) mutations in the Mitogen-activated protein kinase (MAPK) pathway, Neuroblastoma RAS viral oncogene homolog (NRAS) mutations located upstream of the PI3K/AKT/mTOR pathway, the tumor suppressor phosphatase and tensin homolog (PTEN) suppressor gene mutations, p53 tumor suppressor mutations, and PI3K/AKT/mTOR pathway gene mutations [12].

Current therapeutic options for melanoma include surgery, chemotherapy, radiotherapy, immunotherapy, and targeted therapy. Surgery remains the main treatment for early-stage melanoma and commonly includes wide excision and sentinel lymph node biopsy. Chemotherapy may be used for advanced melanoma, although its response rates are limited and systemic toxicities remain a concern. Radiotherapy is mainly used for palliative symptom relief and local tumor control in selected patients [13]. In recent decades, immune checkpoint inhibitors and BRAF/mitogen-activated protein kinase kinase (MEK)-targeted therapies have become central options for metastatic and high-risk melanoma. Immune checkpoint inhibitors enhance antitumor immune responses by blocking inhibitory pathways such as PD-1/Programmed death-ligand 1 (PD-L1), whereas targeted therapies suppress tumor growth in melanoma patients with specific driver mutations, such as BRAF mutations [14,15]. Furthermore, the combination of these treatments represents a promising direction for future research.

### 2.3. Potential Effects of Natural Bioactive Compounds

Medicinal plants and their bioactive compounds may contribute to skin cancer prevention and therapy through multiple mechanisms. For example, triptolide has been reported to induce apoptosis in melanoma cells by inhibiting NF-κB signaling [16]. In preclinical melanoma models, triptolide has also been shown to enhance responses to conventional chemotherapy, including platinum-based agents, suggesting its potential role as a chemosensitizer in combination regimens [17]. In parallel, emerging technologies such as nanotechnology and targeted delivery systems may improve the stability, bioavailability, and skin penetration of natural bioactive compounds [18]. Gene-editing, epigenetic regulation, multi-omics, and artificial intelligence-based approaches may further support target identification, compound optimization, and treatment prediction [19,20]. This is shown in Figure 2.

## 3. Functional Classification of Representative TCM Sources and Bioactive Compounds in Skin Cancer

**Figure 3:**
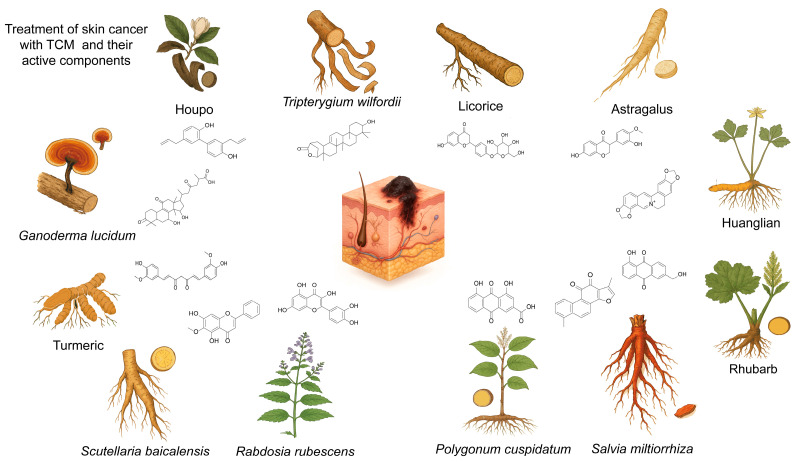
Representative TCM herbs and their bioactive compounds with potential therapeutic effects against skin cancer.

In recent years, TCM-derived natural products have attracted increasing attention as potential sources of anti-skin cancer agents. Owing to their multi-component and multi-targeted characteristics, representative TCM sources can be considered from the perspective of their major biological functions in skin cancer prevention and therapy. In this section, these herbs and their bioactive compounds are discussed in relation to apoptosis and cell-cycle regulation, oxidative stress and metabolic modulation, immune and inflammatory regulation, EMT/metastasis inhibition, and photoprotection-related effects. Figure 3 provides an overview of representative TCM herbs and their bioactive compounds, while Table 1 summarizes their major active ingredients and potential molecular targets.

### 3.1. Apoptosis and Cell-Cycle Regulation

#### 3.1.1. Rhubarb

As a representative anthraquinone-rich TCM source, rhubarb (*Rheum* spp.) is mainly relevant to apoptosis regulation, tumor initiation/promotion, and chemotherapeutic penetration in skin cancer-related studies. Rhubarb mainly refers to medicinal plants such as *Rheum palmatum* L., *Rheum tanguticum* Maxim. ex Balf., and *Rheum officinale* Baill. Among its major bioactive constituents, emodin, aloe-emodin, and chrysophanol are generally regarded as representative anthraquinone compounds [21]. In skin cancer-related research, combined exposure to UVB and aloe-emodin, with ethanol as the vehicle, induced melanin-containing primary cutaneous melanomas in C3H/HeN mice, providing an in situ rodent model for melanoma induction [22]. Aloe-emodin has also been shown to inhibit the growth of non-melanoma skin cancer cells and enhance the skin penetration of 5-FU [23]. Emodin inhibited both the initiation and promotion phases of NOR-1-induced tumors [24].

#### 3.1.2. Turmeric

Turmeric is closely associated with apoptosis and cell-cycle regulation because curcumin and related curcuminoids have been reported to exert antiproliferative, pro-apoptotic, and tumor-delaying effects in skin cancer-related models. Turmeric is the dried rhizome of *Curcuma longa* L. and contains curcuminoids, including curcumin, demethoxycurcumin, and bisdemethoxycurcumin, among which curcumin is the most extensively studied compound in skin cancer-related research [25]. Curcumin inhibited UV radiation-induced skin cancer in SKH-1 mice [26]. It also appeared to inhibit skin cancer formation and prolong tumor onset when administered orally or topically [27]. In melanoma cells, curcumin showed antiproliferative and pro-apoptotic effects in SK-MEL-1 cells [28].

#### 3.1.3. *Rabdosia rubescens*

*Rabdosia rubescens* is mainly relevant to cell-cycle regulation and growth inhibition because its diterpenoid constituent oridonin has been reported to inhibit melanoma proliferation, induce differentiation, and arrest cells at the G2/M phase. Phytochemical investigations have identified diterpenoids, especially oridonin and ponicidin, as the predominant bioactive constituents, together with triterpenoids, flavonoids, phenolic acids, polysaccharides, alkaloids, and other minor components [29]. Oridonin inhibited melanoma cell proliferation and migration, induced differentiation, and arrested the cell cycle at G2/M in K1735M2 cells [30].

#### 3.1.4. *Tripterygium wilfordii*

*Tripterygium wilfordii* represents a source of highly potent apoptosis-regulating compounds, particularly triptolide and celastrol, although their toxicity remains a major translational limitation. Its major bioactive constituents include terpenoids, such as triptolide, tripdiolide, celastrol, and wilforlide A, as well as alkaloids such as wilfordine and related compounds [31]. Several active components of *Tripterygium wilfordii* have shown anti-tumor activity, but their toxicity limits direct clinical translation. Celastrol inhibited mouse B16-F10 melanoma cell survival by modulating the PI3K/AKT/mTOR signaling pathway and suppressing HIF-1α expression [32]. In cutaneous squamous cell carcinoma, triptolide decreased cell viability and migration and promoted apoptosis in A431 and SCL-1 cells. These effects were accompanied by apoptosis/autophagy-associated signaling linked to Akt/mTOR, and the anti-tumor efficacy was further supported in a mouse xenograft model [33].

### 3.2. Oxidative Stress, Metabolic Modulation, and Ferroptosis-Related Effects

#### 3.2.1. *Salvia miltiorrhiza*

*Salvia miltiorrhiza* is associated with oxidative stress regulation, Nrf2 activation, metabolic signaling, and ferroptosis-related tumor suppression through tanshinone-class constituents. It contains lipophilic tanshinones, including tanshinone I, tanshinone IIA, tanshinone IIB, and cryptotanshinone, as well as water-soluble phenolic acids such as salvianolic acids A, B, and C [34]. Tanshinone IIA activated STAT1, promoted PTGS2 expression, and induced ferroptosis, thereby inhibiting melanoma progression [35]. Tanshinones also up-regulated antioxidant defense and cytoprotective genes through Nrf2 transcriptional activation, effectively reducing UV-induced skin damage in Hs27 human dermal fibroblasts and HaCaT keratinocytes [36].

#### 3.2.2. Licorice

Licorice is grouped here because its flavonoids and triterpenoid saponins can regulate oxidative stress, inflammatory signaling, and ferroptosis-related processes in skin cancer-related contexts. Licorice is derived from the dried roots and rhizomes of *Glycyrrhiza uralensis*, *Glycyrrhiza glabra* L., and *Glycyrrhiza inflata* Bat. Phytochemical studies have shown that licorice contains flavonoids, phenolic compounds, polysaccharides, and triterpenoid saponins. Glycyrrhizic acid, a representative triterpenoid saponin, and its metabolite glycyrrhetinic acid are considered key anti-inflammatory and immunomodulatory components [37]. Glycyrrhizic acid can inhibit the release of inflammatory mediators and reduce inflammatory responses [38]. Echinatin has been reported to increase mitochondrial ROS levels and trigger ferroptosis by regulating GSTM3, while also affecting FNR-related proteins and MAPK signaling [39].

#### 3.2.3. *Polygonum cuspidatum*

*Polygonum cuspidatum* is mainly relevant to oxidative stress modulation and photochemoprevention because it is a rich source of resveratrol, a polyphenol with reported effects on apoptosis, proliferation, inflammation, and oxidative stress in NMSC-related models. *Polygonum cuspidatum* contains anthraquinones, flavonoids, phenolic acids, and stilbenes, with resveratrol being one of its most widely studied bioactive compounds [40]. Resveratrol exerts preventive and therapeutic effects against NMSC through multifaceted mechanisms, including apoptosis induction, inhibition of cancer cell proliferation, and modulation of oxidative stress. Its synergistic efficacy in combination with conventional chemotherapeutic agents, together with nanocarrier-based delivery systems, demonstrates considerable promise for enhancing therapeutic outcomes [41].

### 3.3. Immune and Inflammatory Regulation

#### 3.3.1. Astragalus

Astragalus is particularly relevant to immune regulation and treatment resistance because Astragali polysaccharides have been reported to modulate PD-L1-related signaling, chemosensitivity, and T-cell infiltration in melanoma models. Astragalus is derived from the dried roots of *Astragalus membranaceus* (Fisch.) Bunge or *Astragalus membranaceus* var. *mongholicus* (Bunge) P. K. Hsiao. Its major bioactive constituents include flavonoids, astragaloside-type triterpenoid saponins, and polysaccharides [42,43]. Astragali polysaccharides can down-regulate stemness-related genes and enhance the chemosensitivity of drug-resistant melanoma cells, with PD-L1/PI3K/AKT/mTOR signaling potentially involved in this process [44]. Another study showed that Astragali polysaccharides attenuated melanoma tumor sphere formation in vitro and tumorigenicity in vivo, while increasing CD4+ and CD8+ T-cell infiltration in tumor tissues by down-regulating PD-L1 expression [45].

#### 3.3.2. *Ganoderma lucidum*

*Ganoderma lucidum* is placed in the immune and inflammatory regulation category because its polysaccharides and triterpenoids have been associated with UV-related immune modulation, inflammatory regulation, and photoprotective effects. *Ganoderma lucidum* is the dried fruiting body of *Ganoderma lucidum* (Leyss. ex Fr.) Karst. or *Ganoderma sinense* Zhao, Xu et Zhang. Its major bioactive constituents include triterpenoids, such as ganoderic acids, and polysaccharides, particularly β-glucans [46,47]. Ganoderma significantly reversed UV-mediated suppression of dinitrofluorobenzene-induced contact hypersensitivity by increasing CD8+ T cells and reducing CD4+ and FoxP3+ regulatory T cells in the ears of SKH-1 mice [48]. *Ganoderma lucidum* polysaccharides also protected fibroblasts from UVB-induced photoaging [49].

#### 3.3.3. Houpo

Houpo is functionally related to inflammatory regulation and photocarcinogenesis because its major polyphenols, magnolol and honokiol, have been linked to inflammatory mediator regulation, oxidative stress control, and UVB-related signaling. Houpo is the dried bark, root bark, or branch bark of *Magnolia officinalis* Rehd. et Wils. or *Magnolia officinalis* var. *biloba*. Its major active constituents include magnolol and honokiol, together with volatile oils, alkaloids, and flavonoids [50]. Magnolol and honokiol have antibacterial, anti-inflammatory, and antioxidant effects [51]. Magnolol has been reported to induce cell death via PI3K/AKT/mTOR-associated epigenetic modulation in BRAF- and NRAS-mutant melanoma models, suggesting potential utility as an adjunct approach that warrants further in vivo and clinical validation [52]. Honokiol inhibits photocarcinogenesis by targeting UVB-induced inflammatory mediators and cell-cycle regulators [53].

### 3.4. EMT, Invasion, Metastasis, and Drug Resistance

#### 3.4.1. Huanglian

Huanglian is placed in this category because its major alkaloid, berberine, has been closely associated with EMT-related signaling, melanoma cell invasion, and migration. Huanglian refers to medicinal plants from the genus *Coptis* in the Ranunculaceae family, including *Coptis chinensis* Franch., *Coptis deltoidea* C. Y. Cheng et Hsiao, and *Coptis teeta* Wall. Its major bioactive constituents are alkaloids, including berberine, palmatine, jatrorrhizine, coptisine, and epiberberine, together with phenolic acids and other minor compounds [54]. Berberine regulates melanin synthesis by activating PI3K/AKT/mTOR/ERK/GSK3β signaling in B16F10 melanoma cells [55]. It also regulates EMT-related proteins via crosstalk between PI3K/AKT/mTOR/RARα/RARβ, thereby inhibiting melanoma cell invasion and migration in B16 melanoma cells and B16F10 tumor-bearing mice [56].

#### 3.4.2. *Scutellaria baicalensis*

*Scutellaria baicalensis* is included in this category because its flavonoids, particularly baicalein and wogonin, have been reported to regulate invasion-, migration-, and metastasis-related signaling in melanoma models. *Scutellaria baicalensis* Georgi is the dried root of *Scutellaria baicalensis* Georgi and contains multiple small-molecule constituents, among which flavonoids such as baicalin, baicalein, wogonin, chrysin, and oroxylin A are considered major bioactive components [57,58]. *Scutellaria baicalensis* rhizome ethanol extract protected HaCaT cells from H2O2-induced DNA damage and apoptosis by reducing oxidative stress and activating the Nrf2/HO-1 signaling pathway [59]. Wogonin suppressed melanoma aggressiveness by inhibiting B16-F10 cell invasion and migration, with consistent anti-metastatic effects further supported in B16-F10 melanoma-bearing mice [60].

## 4. Mechanisms of Skin Cancer Inhibition by Medicinal Plants and TCM Active Compounds

Natural products derived from TCM contain diverse bioactive molecules with distinct pharmacological activities. In the context of skin cancer, their effects are increasingly understood through specific molecular events involved in tumor initiation and progression, including mitochondrial apoptosis, PI3K/AKT/mTOR-mediated survival signaling, NF-κB-related inflammatory regulation, Nrf2-dependent antioxidant responses, EMT/MMP-associated invasion, and DNA damage repair. Therefore, summarizing these mechanisms at the pathway and molecular-target levels may help clarify how these compounds contribute to skin cancer prevention and treatment. Mechanistically, these signaling changes should be interpreted with appropriate caution. In many preclinical studies, alterations in PI3K/AKT/mTOR, NF-κB, MAPK, ROS/Nrf2, and EMT/MMP-related pathways are mainly inferred from changes in protein expression, phosphorylation status, or tumor-cell phenotypes, rather than from direct target-binding evidence. Thus, these pathways are discussed here as functional regulatory networks involved in apoptosis, cell-cycle control, invasion and metastasis, oxidative stress, inflammation, DNA damage repair, and photoprotection. Such a perspective is important because the same pathway may exert different effects depending on cancer subtype, experimental model, dosage, and treatment context.

### 4.1. Induction of Apoptosis

In recent years, studies have shown that a variety of natural TCM components exhibit significant apoptosis-inducing effects in the treatment of skin tumors by regulating key signaling pathways. In the field of melanoma, Celastrol induces apoptosis by activating the ROS-dependent mitochondrial pathway and inhibiting PI3K/AKT/mTOR signaling (in vitro, B16 melanoma cells) [61]. In a separate in vivo study, antitumor activity was achieved by targeting interleukin-2 (IL-2) signaling specifically by inhibiting IL-2 binding to CD25 and thereby modulating T-cell mediated immune responses (in vivo, IL-2 dependent mouse model) [62]. Triptolide also exhibits multi-target effects. In the cutaneous squamous cell carcinoma model, triptolide simultaneously induces apoptosis and autophagy through the PI3K/AKT/mTOR pathway [33]. In melanoma A375 cells, triptolide induces S-phase block by regulating the cyclin E/CDC25A axis. It exerts anti-tumor effects in coordination with the Caspase-mediated mitochondrial apoptotic pathway [63]. Research on the anti-melanoma mechanism of isoliquiritigenin has also progressed. Xiang et al. found that this component inhibits cell proliferation by regulating the miR-27a/POU2F3/c-MYC/p53 signaling axis while blocking the epithelial–mesenchymal transition (EMT) process, and shows significant apoptosis-inducing effects (in vitro, A2058, B16, and A375 cells; in vivo, A2058 xenograft in nude mice) [64]. It is worth noting that although Ponicidin has been shown to possibly inhibit melanoma growth through the NF-κB signaling pathway, its specific target of action and downstream effector molecules still need to be deeply analyzed [65]. In contrast, cryptotanshinone induces apoptosis through the ROS-mitochondrial apoptosis pathway by inhibiting proliferation and blocking cell migration and invasion [66].

In the study of squamous cell carcinoma of the skin, Hao et al. confirmed that resveratrol initiates apoptotic program in tumor cells by upregulating the transcription and translation levels of the *p53* gene and suppressing the expression of survivin (SVV) at the same time through the establishment of the A431 xenograft model [67]. In addition, Emodin, as another important active ingredient, its pro-apoptotic effect may be realized through the dual mechanism of mitochondrial endogenous pathway and death receptor exogenous pathway, but the specific target of its action is still to be elucidated [68]. A recent study further showed that a flavonoid-rich extract of *Dalbergia odorifera* inhibited proliferation and migration and promoted apoptosis in cSCC, accompanied by down-regulation of Bcl-2 and up-regulation of Bax (in vitro, A431 cells; in vivo, DMBA/croton oil-induced cSCC mouse model) [69].

Taken together, apoptosis-related studies suggest that several natural compounds act by shifting the balance between pro-survival signals and mitochondrial or death receptor–associated apoptotic execution. Celastrol and triptolide are among the most consistently investigated compounds, with evidence from both melanoma and cSCC models. However, the current evidence mainly supports apoptosis-associated pathway modulation rather than a unified primary target. Future studies should therefore move beyond measuring caspase activation or apoptosis-related protein expression and include genetic rescue, target-engagement assays, and dose–response comparisons across skin cancer subtypes to determine whether apoptosis is a primary mechanism or a downstream consequence of broader cellular stress.

### 4.2. Inhibition of Growth and Metastasis

**Figure 4:**
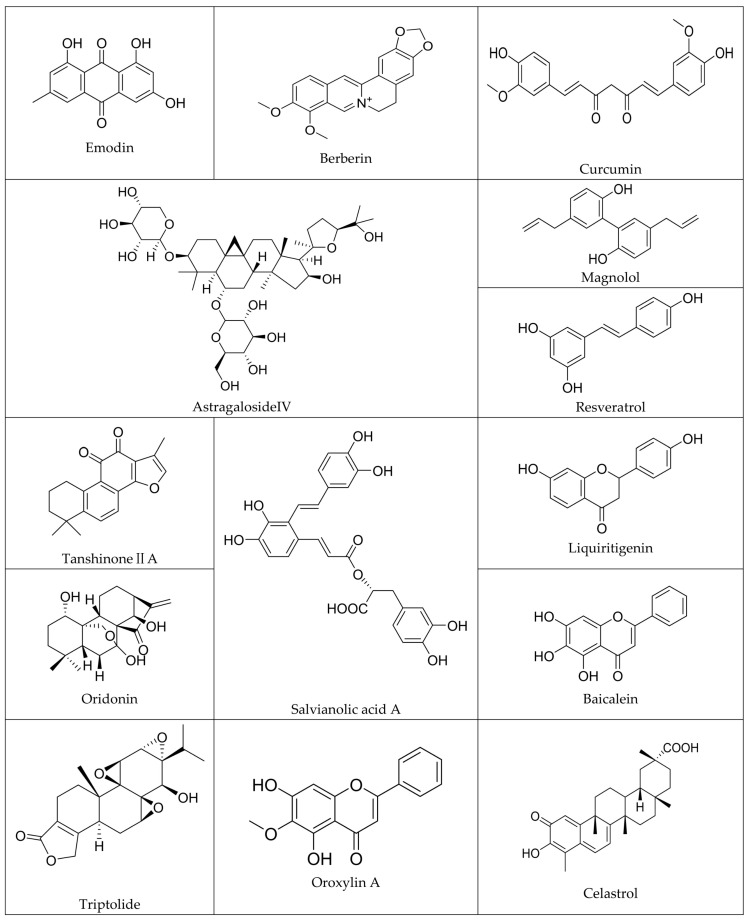
Structural diagrams of representative active components from medicinal plants and TCM.

The molecular mechanisms of various TCM active compounds in skin cancer treatment have been gradually revealed. The chemical structures of representative active compounds discussed in this section are shown in Figure 4. Triptolide significantly inhibited melanoma cell proliferation by inhibiting the Src-ERK signaling pathway, and its antitumor effect was verified (in vitro, SK-MEL-5 and SK-MEL-28 cells; in vivo, SK-MEL-5 subcutaneous xenograft in BALB/c nude mice) [70]. Triptolide further interfered with the self-repair process of tumor cells by inducing DNA damage and inhibiting the mRNA expression of DNA repair-related genes (in vitro, A375 cells) [71].

Baicalein inhibited the migration and invasive ability of B16F10 cells by down-regulating the expression of NF-κB (in vitro, B16F10 cells) [72]. The anti-melanogenic effect of baicalein was associated with the activation of ERK signaling pathway, and reduced tyrosinase synthesis through the down-regulation of MITF (in vitro, B16F10 cells) [73]; whereas Baicalein not only inhibited the expression and activity of MMP-2/-9, and blocked the PI3K/AKT/mTOR signaling pathway to reduce the aggressiveness of B16F10 cells [74], but also inhibited tumor cell migration and aggressiveness through the modulation of the mTOR-HIF-1 α pathway to inhibit glucose metabolism in tumor cells, thereby inhibiting tumor growth in vivo (in vitro, B16F10 and A375 cells) [75].

Isoliquiritigenin acts through a dual mechanism: on the one hand, isoliquiritigenin may restrict cell proliferation by inhibiting miR-301b and inducing its target LRIG1 (in vitro, A2058 and A375 cells; in vivo, immunodeficient mice) [76]; On the other hand, Isoliquiritigenin targets the histone H2A.Z variant 1 (H2A.Z.1)–E2F transcription factor 1 (E2F1) pathway to hinder cell-cycle progression and DNA damage repair, ultimately inhibiting melanoma metastasis (in vitro, A375 and SK-MEL-28 cells) [77].

Among the activating components contained in *Salvia miltiorrhiza*, tanshinone IIA inhibited the proliferation of A375 cells by activating autophagy-related genes (Beclin-1, LC3-II) and blocking the PI3K/Akt/mTOR/p70S6K1 pathway [78]; and cryptotanshinone inhibited mTOR-mediated HIF-1α expression via the AMPK pathway, thereby reducing the activity of the glycolytic rate-limiting enzyme PFK and inducing cell-cycle arrest and apoptosis (in vitro, B16F10 and A375 cells; in vivo, C57BL/6N mice and BALB/c nude mice) [79]. At the same time, cryptotanshinone inhibited the mTORC1 signaling pathway, down-regulated the expression of cyclin D1 and inhibited the phosphorylation of Rb, which induced the cell cycle to block at the G_0_/G_1_ phase, and thus inhibited the proliferation of cancer cells (in vitro, Rh30 cells) [80]. Tanshinol exerts antitumor activity against melanoma by regulating miR-1207-5p/CHPF signaling (in vitro, A375 cells) [81]. In addition, Salvianolic Acid B significantly inhibits melanoma migration by directly binding to β-actin (in vitro, A375 and B16 cells) [82], whereas Salvianolic Acid A regulates the G_2_/M phase checkpoint through the Chk2-Cdc25A-Cdc2 pathway (in vitro, A375 and A2058 cells) [83].

Other constituents such as Honokiol exerted anti-melanoma cancer cells by attenuating the PI3K/AKT/mTOR and Notch pathways of rapamycin (in vitro, B16F10 cells) [84]; *Astragalus membranaceus* polysaccharides enhanced the inhibitory effect of immune checkpoint inhibitors on lung metastatic melanoma in mice [85]; and *Astragalus membranaceus* extracts significantly activated the ERK signaling pathway to regulate melanin synthesis (in vitro, B16F10 cells) [86]. Curcumin inhibits the progression of squamous skin cancer by inhibiting pS6 [87], while curcumin enhances miR-222-3p levels to reduce SRY (sex determining region Y)-box 10 (SOX10) expression and ultimately inactivates the Notch pathway in melanoma to inhibit growth and metastasis [88], and Aromatic-turmerone inhibits melanogenesis by inactivating the CREB/MITF pathway (in vitro, B16F10 cells) [89]. Calycosin exhibit anti-melanogenic activity by regulating PKA/CREB and p38 MAPK-mediated microphthalmia-associated transcription factor (MITF) down-regulation, thereby inhibiting melanin synthesis (in vitro, B16F10 cells; in vivo, zebrafish embryos) [90]. Oridonin alba blocked epithelial–mesenchymal transition (EMT) by inhibiting PI3K/AKT/mTOR, GSK-3β phosphorylation in the presence of TGF-β, thereby inhibiting melanoma migration and invasion (in vitro, B16F10 and A375 cells) [91]. Curcumol inhibited ERK/NF-κB signaling and promoted miR-152-3p expression to inactivate the c-MET/PI3K/AKT/mTOR signaling pathway (in vitro, B16 cells) [92].

**Figure 5:**
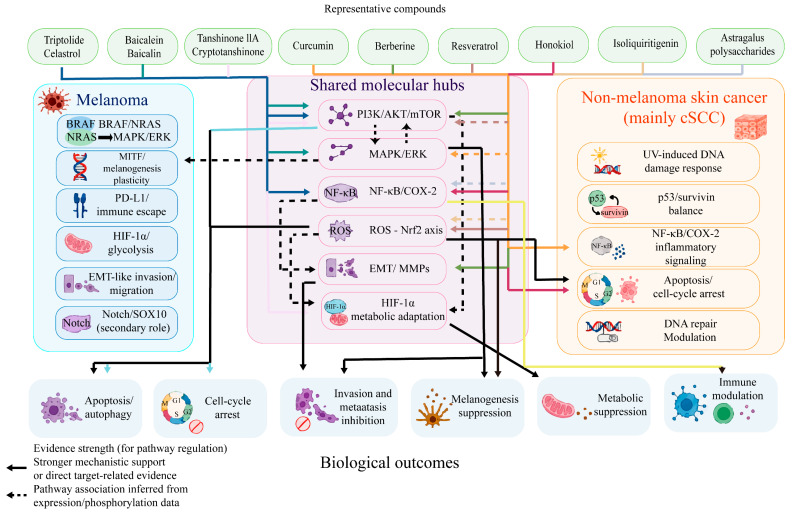
Signaling pathways regulated by active components from medicinal plants and TCM. The colors of the arrows correspond to the respective TCM-derived compounds listed at the top of the figure.

Ginsenoside Rf specifically reduced melanogenesis-related gene expression by inhibiting the PKA/CREB/MITF pathway (in vitro, Mel-Ab and HEK-293T cells) [93]. Ginsenoside Rg3 inhibits ERK and Akt signaling pathways, induces cell cycle arrest in S-phase, down-regulates matrix metalloproteinases MMP-2 and MMP-9 to inhibit metastasis, and reduces the expression of vascular endothelial growth factor (VEGF) to inhibit melanoma proliferation in multiple dimensions (in vitro, B16 cells) [94]. Ginsenoside Rh2 inhibited SCC growth by reducing Lgr5-positive CSCs through modulating the interaction of autophagy and β-catenin signaling [95], whereas ginsenoside Re inhibited melanin synthesis through the AKT/ERK signaling pathway-mediated degradation of the ubiquitin-proteasome pathway, which in turn down-regulated the expression of MITF target genes (in vitro, B16F10 cells; in vivo, zebrafish AB wild-type and C57BL/6 mice) [96]. These studies systematically reveal the potential of natural ingredients to synergistically inhibit the development of skin cancer through multi-targets and multi-pathways, as shown in Figure 5.

Overall, the evidence summarized in this subsection indicates that many natural compounds suppress tumor growth, migration, or invasion through coordinated effects on cell-cycle regulation, cytoskeletal remodeling, metabolic adaptation, and extracellular matrix degradation. Nevertheless, anti-proliferative and anti-metastatic activities should be distinguished carefully. Reduced cell viability can secondarily decrease migration, and wound-healing or transwell assays cannot fully model the multistep process of metastasis in vivo. Therefore, compounds with promising anti-invasive effects should be further evaluated using three-dimensional tumor models, orthotopic or immune-competent animal models, and metastasis-specific endpoints. This would help determine whether the observed effects reflect true suppression of metastatic dissemination or indirect consequences of impaired growth and survival.

### 4.3. Anti-Inflammatory and Antioxidant

The TCM active compounds can inhibit multidimensional mechanisms of skin tumors by regulating oxidative stress and inflammatory responses. Curcumin enhances mitochondrial autophagy and function, reduces oxidative stress, and suppresses inflammatory factors such as IL-18 by targeting YAP1, ultimately alleviating UVB-induced skin photodamage (in vitro, HaCaT keratinocytes; in vivo, UVB-induced skin injury model in C57 mice) [97]. Coptisine protects HaCaT keratinocytes by activating Nrf2 signaling, thereby reducing oxidative stress-related DNA damage and apoptosis (in vitro, HaCaT cells) [98]. Notably, tanshinone-I and dihydrotanshinone may inhibit UVA-induced melanogenesis by enhancing the Nrf2/ARE pathway activity, up-regulating the expression of antioxidant enzymes and decreasing the ROS level (in vitro, HEM cells and Nrf2-knockdown HEM cells; UVA exposure) [99]. The volatile oil from ginger in ginger synergistically enhanced the antioxidant defense system of B16 melanoma cells by elevating GSH, SOD and CAT activities (in vitro, B16 melanoma cells) [100].

In the study of anti-inflammatory mechanisms, *Ganoderma lucidum* extract exerted anti-tumor effects by inhibiting the release of pro-inflammatory factors such as IL-6 and IL-8 and matrix metalloproteinases (MMP-2 and MMP-9) (in vitro, B16-F10 cells) [101]. Polydatin effectively inhibited UVB-induced inflammatory responses by modulating COX-2 expression (in vitro, HaCaT keratinocytes) [102]. Notably, Magnolol effectively blocked the inflammation-tumor transformation pathway through dual inhibition of iNOS and COX-2 gene expression (in vivo, TPA-induced skin inflammation/tumor model in ICR mice) [103].

These studies systematically illustrate how natural products build a complex regulatory network by targeting key hubs of oxidative stress (e.g., Nrf2, NOX) and inflammation (e.g., NF-κB, COX-2), which converge to mitigate skin tumor progression. By modulating these pathways, compounds like baicalin, liquiritin, and polydatin show significant promise in skin cancer prevention and treatment, offering a solid theoretical foundation for the development of novel photoprotective and anticancer strategies. The antioxidant and anti-inflammatory activities of natural compounds should be interpreted in a context-dependent manner. Nrf2 activation and ROS reduction may protect normal keratinocytes from UV-induced damage, whereas excessive ROS generation can contribute to tumor-cell apoptosis. Therefore, future studies should clearly distinguish chemopreventive effects in normal or premalignant skin from cytotoxic effects in established tumor cells.

### 4.4. DNA Damage Repair and Photoprotection

**Figure 6:**
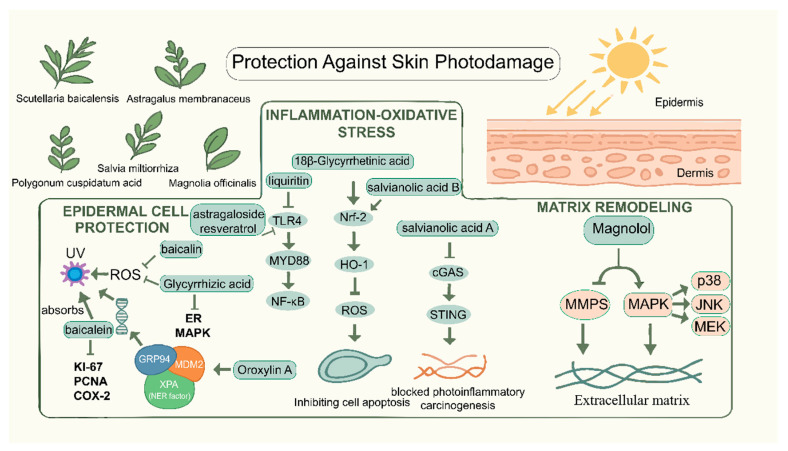
Photoprotective effects of TCM-derived bioactive compounds against UV-induced skin damage. Arrows indicate promotion, while T-shaped symbols indicate inhibition.

The representative photoprotective mechanisms of TCM-derived bioactive compounds against UV-induced skin damage are summarized in Figure 6. Studies on the molecular regulatory mechanisms of photosensitive skin damage have revealed that a variety of plant active ingredients exert synergistic protective effects by targeting key signaling pathways. In terms of epidermal cell protection, baicalein exhibits direct photoprotective capacity by absorbing UVB radiation (in vitro, HaCaT cells) [104]. In parallel, baicalin provides complementary protection: it significantly attenuates chronic photodamage in C57BL/6 mice by suppressing proliferative markers (e.g., Ki-67 and PCNA) and inflammatory mediators such as COX-2 (in vivo, C57BL/6 mice) [105]. Moreover, by scavenging ROS and strengthening antioxidant defenses, baicalin effectively alleviates UVA-induced apoptosis (in vitro, human skin fibroblasts) [106]. Glycyrrhizic acid has a potent anti-photodamage effect by quenching ROS, preventing calcium imbalance, and then inhibiting ER stress and activation of MAPK pathways (p38, JNK, MEK), which ultimately attenuated mitochondrial damage and cell apoptosis (in vitro, Hs68 cells) [107]. Oroxylin A extracted from *Scutellaria baicalensis* stabilizes XPA protein and enhances nucleotide excision repair (NER) by modulating GRP94/MDM2-XPA binding, providing a unique molecular protective mechanism against UVB injury (in vivo, SKH-1 mice) [108].

In the field of inflammation-oxidative stress interactions, liquiritin exhibits protective effects through dual mechanisms: on the one hand, liquiritin inhibits the release of pro-inflammatory factors through the TLR4/MyD88/NF-κB signaling axis, and on the other hand, it dynamically regulates redox homeostasis to inhibit oxidative stress to prevent UVB-induced skin damage (in vitro, HaCaT cells; in vivo, SKH-1 mice) [109]. 18β-Glycyrrhetinic acid promotes Nrf2 nuclear translocation through activation of the Nrf2/HO-1 signaling pathway, reduces ionizing radiation-induced reactive oxygen species (ROS) accumulation in HaCaT cells, and inhibits apoptosis (in vitro, HaCaT cells) [110]. Salvianolic acid A prevents skin damage caused by ultraviolet radiation by inhibiting cGAS STING activation (in vitro, HaCaT cells and THP-1 cells; in vivo, female BALB/c mice, C57BL/6J mice, and STING^−/−^ knockout mice) [111], whereas salvianolic acid B focused on activating the nuclear factor erythroid 2-related factor 2 (Nrf2) pathway to antagonize photo-aging processes (in vitro, human dermal fibroblasts (HDFs); in vivo, UVB-induced skin photoaging in athymic mice) [112]. It is worth noting that although astragaloside and resveratrol both target the TLR4/NF-κB system, their dimensions of action differ: the former focuses on keratinocyte protection (in vitro, HaCaT cells), while the latter exerts a broad-spectrum photopreventive effect through this pathway (in vitro, Normal Human Epidermal Keratinocytes) [113,114].

As for the regulation of matrix remodeling, Magnolol demonstrated its unique advantage through multi-targeted intervention: it not only inhibits the activities of matrix degrading enzymes such as MMP-1, -9 and -13, but also regulates the MAPK signaling network to form a multidimensional anti-photodamage barrier (in vivo, HR-1 hairless mice) [115].

The studies in this subsection underscore the importance of DNA repair, particularly NER/XPA-related repair, and Nrf2-mediated oxidative stress management in protecting skin cells from UV-induced damage. These photoprotective effects are associated with several molecular processes discussed above, including ROS regulation, UV-related DNA damage response, MAPK/NF-κB-mediated inflammatory signaling, cGAS-STING pathway activation, and MMP-associated matrix remodeling. Natural compounds such as baicalin, oroxylin A, glycyrrhizic acid, liquiritin, and salvianolic acids may therefore mitigate photoaging, DNA damage, and inflammation through multiple converging pathways. Despite promising preclinical evidence, further investigations into their translational potential and clinical application are still needed for optimizing photoprotective strategies. These findings suggest that the photoprotective effects of TCM-derived compounds are more closely related to skin cancer prevention than to direct treatment of established tumors. Compounds that reduce UV-induced ROS accumulation, inflammatory activation, or DNA damage may help interrupt early photocarcinogenic events, but such effects do not necessarily indicate tumor-regressive activity. Therefore, photoprotection-related evidence should be interpreted separately from therapeutic evidence based on tumor growth, invasion, metastasis, or survival outcomes.

## 5. Combination of Natural Bioactive Compounds

In terms of antioxidant defense and photoprotection, the combined application of rutin and ascorbic acid significantly enhances the anti-apoptotic ability of skin keratinocytes and fibroblasts against UVA/UVB radiation through synergistic scavenging of free radicals [116]. This synergistic mode of enhancement was similarly reflected in the combination of baicalin with other antioxidants to form a multilevel photodamage protection network.

In the field of apoptosis pathway regulation, multi-component synergistic effects are particularly prominent, as Gobika Arumugam’s team found that the binary combination of resveratrol, epigallocatechin-3-gallate, and diallyl trisulfide regulates the Bcl-2 family of proteins through dual regulation of the pro-apoptotic protein Bax/Bad, while inhibiting the anti-apoptotic protein Bcl-2, which activates the caspase-3/-9 cascade reaction, and the cascade reaction through the mitochondria. 9 cascade reaction, which significantly enhanced the apoptotic effect of A431 skin cancer cells through the mitochondrial pathway [117]. Similarly, the combination of α-santalol with magnolol and honokiol effectively inhibited the proliferative viability of tumor cells despite a 50% reduction in the administered dose through a dose synergistic effect, demonstrating highly effective chemopreventive potential [118].

In chemotherapeutic sensitization studies, the combination of natural products with traditional chemotherapeutic drugs has shown significant advantages, and the combination of 5-FU and honokiol not only enhances cell growth inhibition, but also produces synergistic antitumor effects through the activation of apoptotic pathway [119]. The combination of Radix Astragali and Tanshinone significantly enhanced the cytotoxicity of carboplatin in B16 melanoma cells by interfering with the PI3K/AKT/mTOR signaling axis, which provided a new idea to overcome the resistance to chemotherapy [120]. The presence of triptolide in carboplatin treatment selectively inhibited NER pathway activity, suppressed A375 and B16 cell viability, migration, invasion and induced apoptosis and significantly inhibited tumor progression in nude mice by inhibiting cell proliferation and inducing apoptosis [17].

The study of signaling pathway co-regulation mechanism revealed that resveratrol could enhance the tumor cell killing effect of photodynamic therapy by activating the p38/MAPK pathway [121]. The combination of berberine and doxorubicin reduced the toxicity of doxorubicin and enhanced the inhibitory effect of doxorubicin on cell growth and proliferation [122]. This multimodal therapeutic strategy opens up new avenues for the treatment of advanced skin cancer. These studies have systematically elucidated the synergistic/additive effects of natural product combinations through the spatial and temporal differences in the regulation of apoptotic pathways, metabolic networks and signaling systems, which may inform the development of combination regimens with a potentially improved therapeutic index, although toxicity reduction has primarily been demonstrated in preclinical settings.

## 6. Application of Natural Bioactive Compounds with New Technology

### 6.1. Delivery Strategies and Formulation Innovations

To provide a systematic analysis of delivery strategies for skin cancer, we summarized technologies according to the key biological barriers they are designed to overcome. In practice, these barriers span limited penetration across the stratum corneum, inadequate stability and bioavailability of labile phytochemicals, insufficient tumor targeting and cellular uptake, and the need for co-delivery to achieve synergistic effects. Organizing the literature in this way allows cross-platform comparison and helps pinpoint gaps that continue to hinder translation (Table 2).

In lipid carrier technology, a topical nanostructured lipid carrier gel optimized for quercetin and resveratrol achieved synergistic delivery of both agents, enhancing transdermal permeation and increasing epidermis–dermis deposition/penetration depth (ex vivo, excised rat skin), and showing no detectable dermal irritation (in vivo, Swiss albino mice); these benefits were attributed to nanoscale surface effects, lipid carrier–mediated stratum corneum permeation enhancement, and high encapsulation efficiency enabling efficient drug loading and release [123]. Similarly, a nanoemulsion system for quercetin was developed that enhanced skin permeability and effective skin retention, thereby enhancing its anti-cancer prospects [124]. Novel curcumin-resveratrol solid nanoparticles synergistically inhibit melanoma cell proliferation, and the use of a delivery system loaded with solid lipid nanoparticles (SLNs) enhances the skin penetration and anticancer efficacy of these polyphenol combinations [125]. Hypermorphic liposome technology has been innovatively utilized to achieve the co-encapsulation of resveratrol and 5-fluorouracil, thereby altering the action of 5-fluorouracil and increasing the activity of resveratrol. This synergistic delivery system significantly enhanced the anti-skin cancer activity by modulating cell cycle block (G_1_/S phase) [126]. In a preclinical B16F10 melanoma model (in vitro, B16F10 cells; in vivo, tumor-bearing C57BL/6 mice), Celastrol-loaded nanoparticles improved the therapeutic index, with enhanced anti-melanoma activity and reduced systemic toxicity indicators versus free celastrol, including stable body weight, no evident H&E organ lesions, and lower off-target organ accumulation alongside tumor-biased biodistribution [127]. While lipid-based carriers have shown consistent success in improving dermal penetration and enhancing bioavailability of lipophilic compounds, challenges such as scalability and long-term stability remain. Additionally, comparisons with other delivery systems like nanogels and liposomes, which offer greater controlled release profiles, are limited and need further evaluation.

In the field of nanofiber technology, an electrostatically spun nanofiber system with multifunctional bioactive anticancer and antimicrobial activity has been developed, demonstrating multiple advantages: emodin-loaded nanofiber dressings not only exhibit excellent wettability, high encapsulation efficiency, and biphasic release kinetics, but also activate tumor cell apoptotic pathways by modulating MDA and ROS levels [128]. Another research team designed polycaprolactone nanofibers combining tanshinone IIA with bioactive polymers, creating a new paradigm of synergistic therapy combining phytotherapeutics, nanomedicine delivery and bioactive polymer functionalization [129]. Nanofibers exhibit great potential for drug release and activating apoptotic pathways, but their efficacy in tumor targeting and skin retention need to be improved. Future research should focus on optimizing nanofiber surface modification and enhancing tumor-targeting capabilities for clinical use.

Several studies have demonstrated differentiated technology pathways for the optimization of curcumin delivery: Curcumin-containing chitosan nanogels showed a 4-fold increase in steady-state transdermal flux of curcumin compared to control curcumin solutions and loosening of the epidermal stratum corneum, which facilitated penetration [130]. Bioadhesive membranes made of curcumin-containing chitosan showed compatibility with dermal applications with controlled drug release and enabled drug penetration into deeper layers of the skin, ensuring localized distribution of the phytochemicals. The amount of drug that penetrates the tissue will effectively reduce cell viability in the metastatic tumor lineage beyond the response achieved with a single dose of radiation therapy [131].

In contrast, the curcumin-containing liposome–siRNA complex system utilized the lattice protein-mediated endocytosis pathway to achieve efficient co-delivery, and its synergistic effect with STAT3 siRNA significantly enhanced apoptosis induction, and co-delivery of curcumin and STAT3 siRNA using liposomes resulted in significantly increased growth inhibition and apoptotic events in cancer cells, as compared to treatment with pure curcumin and free STAT3 siRNA [132].

Carboxymethylcellulose-casein nanogels: Layer-by-layer encapsulation of casein and folic acid (CA/FA) for targeted delivery of curcumin for the treatment of skin cancer showed higher stability, higher cellular uptake leading to enhanced cytotoxicity and apoptotic effect on MEL-39 melanoma cancer cells overexpressing folate receptor [133]. Synthesis of novel TPGS-stabilized PLGA nanopatterned films containing curcumin, on the other hand, significantly enhanced cytotoxicity against epidermal cancer cells through surface topology optimization [134].

In the application of natural osmotic enhancers, nano-emulsion systems modified with glycyrrhizin effectively enhanced the anti-melanoma activity of 5-fluorouracil [135]. Solid lipid nanoparticle technology has been shown to significantly increase the cellular uptake efficiency of resveratrol compared with conventional passive diffusion, providing an important rationale for improving the bioavailability of lipophilic ingredients [136].

Innovative formulation techniques have led to the development of a new ultrasonic emulsification method for the preparation of optimized nanostructured lipid carrier gels containing 5-fluorouracil and resveratrol, which has been shown to significantly improve and sustain drug release, and to achieve markedly higher efficacy of the combination flax gel against the A431 cell line compared with conventional formulations; additional studies have further confirmed its efficacy through in vivo experiments [137,138]. The berberine-encapsulated targeted delivery system, on the other hand, utilized the active targeting properties of phenylboronic acid-branched starch nanoparticles to demonstrate superior penetration and apoptosis-inducing effects in a 3D tumor sphere model [139].

Structural modification of natural products in terms of methoxylation of magnolol/honokiol significantly enhanced their skin absorption, maximized skin targeting and minimized transdermal penetration [140]. Furthermore, by targeting the structure of thujaplicins the methoxylated derivative (2-O-Methylmagnolol) could enhance the pro-apoptotic effect by modulating lncRNA GAS5 expression [141].

**Figure 7:**
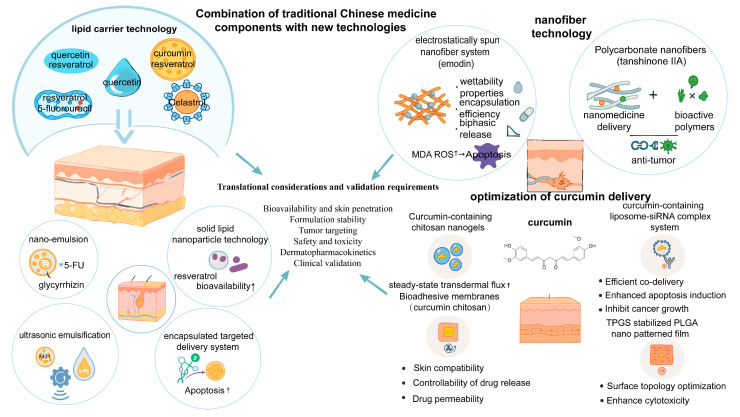
Combination of natural bioactive compounds with emerging technologies. The blue arrows indicate that these delivery strategies and new technologies are linked to the translational considerations and validation requirements shown in the center.

These technological breakthroughs provide diverse solutions for the construction of transdermal antitumor agents with clinical translational potential, fully reflecting the development trend of synergistic innovation between nanotechnology and natural active ingredients, as shown in Figure 7.

Several challenges need to be overcome for the successful clinical translation of TCM-derived therapies for skin cancer. Key barriers include mass manufacturing issues, especially with lipid carriers and nanofibers. Standardizing production techniques and quality control processes will be essential for wider clinical application [142]. Another challenge is the lack of standardized dermatokinetic evaluations, such as skin retention, penetration, and bioavailability, which are crucial for assessing the effectiveness of these delivery systems [143]. Safety concerns, particularly related to skin penetration enhancers and co-delivery systems, also remain [144]. While promising in preclinical studies, more long-term toxicity data are needed, particularly regarding immune-related toxicity [145].

Future research should focus on improving tumor targeting by optimizing delivery systems for better tumor specificity. Additionally, personalized therapies based on genomic profiling and co-delivery systems for synergistic effects are likely to play a significant role in advancing TCM-based treatments. Finally, clinical trials are needed to evaluate the long-term safety and efficacy of these formulations in skin cancer treatment [146].

### 6.2. Safety, Metabolism, and Clinical Translation Considerations

Although natural bioactive compounds show promising anti-skin cancer activity in preclinical models, their clinical translation requires a careful assessment of safety, metabolism, and therapeutic window. This issue is particularly important for highly potent compounds such as triptolide and celastrol. Triptolide has been associated with a narrow therapeutic window and multiple organ toxicities, especially hepatotoxicity and nephrotoxicity, and its hepatotoxicity has been linked to CYP450 enzymes, P-glycoprotein, oxidative stress, excessive autophagy, apoptosis, metabolic disorders, immune responses, and gut microbiota-related mechanisms [147]. Celastrol also exhibits potent anticancer activity, but its poor water solubility, short half-life, low bioavailability, narrow therapeutic window, and potential systemic toxicity limit direct clinical application [148]. Therefore, these compounds should not be regarded simply as “natural and safe” agents; rather, their anticancer potency should be balanced against systemic exposure, organ-specific toxicity, and possible herb–drug interactions.

Metabolism is another key factor affecting translational feasibility. After topical administration, natural compounds may undergo local biotransformation in the epidermis and dermis through cutaneous drug-metabolizing enzymes, including phase I and phase II enzymes [149]. The fraction that penetrates into systemic circulation may subsequently undergo hepatic metabolism, including oxidation, glucuronidation, sulfation, and glutathione-related conjugation. For example, curcumin is limited by poor absorption, rapid metabolism, and rapid systemic clearance, whereas resveratrol is also characterized by low oral bioavailability and rapid first-pass metabolism [150,151]. Therefore, future formulation studies should evaluate not only tumor-cell cytotoxicity, but also skin pharmacokinetic and systemic pharmacokinetic parameters, including skin retention, transdermal flux, local irritation, systemic exposure, hepatic metabolism, and long-term organ toxicity. The evidence level, major molecular hubs, safety and metabolism concerns, and translational status of representative natural bioactive compounds and formulations are summarized in Table 3.

ClinicalTrials.gov data suggest that the clinical translation of natural-product-based formulations in skin-related indications remains at an early stage. A phase 1 study has evaluated a natural-product-containing bioadhesive nanoparticle sunscreen for preventing UVR-induced cellular and DNA damage in human skin, indicating translational interest in photoprotection and skin cancer prevention [152]. In addition, topical resveratrol gel has been registered for melasma [153], oral curcumin has been evaluated for radiation dermatitis [154], and liposomal curcumin has entered clinical trials in cancer patients [155]. However, these studies are mainly related to photoprotection, non-malignant skin conditions, radiation-induced skin injury, or non-dermatological cancers. Direct clinical evidence supporting curcumin- or resveratrol-based nanopreparations for the treatment of established skin cancer remains limited. Future studies should therefore integrate formulation safety, skin pharmacokinetic evaluation, toxicology, and well-designed clinical trials in defined skin cancer populations. The heterogeneity of skin cancer also limits direct translation from preclinical models. Because melanoma, SCC, and BCC differ in molecular drivers, immune context, metastatic potential, and treatment sensitivity, results obtained from a single cell line or xenograft model should be interpreted cautiously. Broader validation is needed to distinguish generalizable mechanisms from model-specific findings.

## 7. Conclusions

Natural bioactive compounds show considerable potential for skin cancer prevention and therapy because of their multi-component, multi-target, and pathway-modulating properties. Available preclinical evidence suggests that TCM-derived compounds may exert anti-skin cancer effects by inducing apoptosis, blocking cell-cycle progression, suppressing angiogenesis and metastasis, regulating immune and inflammatory responses, reducing oxidative stress, and enhancing photoprotection. However, most current evidence is derived from in vitro experiments and animal models. Systematic toxicology, pharmacokinetic characterization, skin pharmacokinetic evaluation, and clinical validation remain insufficient, particularly for potent compounds with narrow therapeutic windows. In addition, the complex composition of TCM, limited standardization of active constituents, poor bioavailability, and insufficient tumor targeting continue to restrict clinical translation.

To address the limitations of TCM and their active components in skin cancer research, these agents can be combined with emerging technologies. With the help of nanocarrier technology or exosome-targeted delivery system, the stability of active ingredients and tumor tissue-specific enrichment can be significantly improved; combined with epigenetic means, it is expected to reveal the regulation of key genes of skin cancer by TCM ingredients. In addition, exploring the combination of active ingredients of TCM with photodynamic therapy and immune checkpoint inhibitors, or optimizing their pharmacodynamic properties through structural modification and formulation innovation may become a new direction to increase the efficacy and potentially enhance tolerability.

## Figures and Tables

**Table 1:** Representative TCM sources, major bioactive compounds, and potential molecular targets relevant to skin cancer.

TCM	Main Active Ingredients	Potential Targets
Rhubarb	emodin, aloe-emodin, chrysophanol, rhein	TNF-α ↑, caspase-3/8/9 ↑, p53 ↑, ROS ↑, Bax ↑, Bcl-2 ↓
Huanglian	berberine, coptisine, epiberberine, palmatine	PI3K/AKT/mTOR ↓, MITF ↓, E-cadherin ↑, Bax ↑, Bcl-2 ↓, Nrf2/HO-1 ↑
Turmeric	curcumin, demethoxycurcumin, gallic acid, protocatechuic acid, chlorogenic acid	NF-κB/AP-1 ↓, COX-2 ↓, TNF-α ↓, Nrf2 ↑, ROS ↓, PI3K/AKT/mTOR ↓, miR-222-3p ↑, Notch ↓
Astragalus	Astragalus, astragalosides I-IV, calycosin, Narcissoside	PD-L1 ↓, PI3K/AKT/mTOR ↓, ERK ↑, TLR4 ↓
*Ganoderma lucidum*	ganoderic acid A/B/C, ganoderiol, ganoderal, ganoderma lucidum polysaccharides	ROS ↓, MMPs ↓, CD8+ T ↑, CD4+ T ↓ IL-6/IL-8 ↓, MMP-2/MMP-9 ↓
Houpo	magnolol, honokiol, magnoflorine, β-eudesmol, α-eudesmol	PI3K/AKT/mTOR ↓, NF-κB/COX-2 ↓, IL-6 ↓, ROS ↓, TNF-α ↓, Cyclin D1/CDK4/6 ↓, p21/p27 ↑
*Polygonum cuspidatum*	resveratrol, emodin, rhein, rutin, Kaempferol, gallic acid, caffeic acid	NF-κB ↓, TNF-α ↓, IL-6 ↓, ROS ↓, MMP-2/MMP-9 ↓
*Salvia miltiorrhiza*	salvianolic acid A/B/C, tanshinone I, tanshinone IIA, cryptotanshinone	STAT1 ↑, COX-2 ↑, Nrf2 ↑, HO-1 ↑ NQO1 ↓, ROS ↓, Bax ↑, Bcl-2 ↓, caspase-3 ↑, PI3K/AKT/mTOR ↓, HIF-1α ↓
*Rabdosia rubescens*	oridonin, ponicidin, Quercetin, Kaempferol, luteolin	caspase ↑, MMP-2/MMP-9 ↓, NF-κB ↓, PI3K/AKT/mTOR ↓, GSK-3β ↓, EMT ↓
Licorice	liquiritin, isoliquiritigenin, glabridin, liquiritigenin, glycyrrhizin	GSTM3 ↓, ROS ↑, FNR ↓, MAPK ↓, Nrf2/HO-1 ↑, TLR4/MyD88/NF-κB ↓, ER ↓, miR-301b ↓, H2A.Z.1–E2F1 ↓, EMT ↓, Bax ↑, Bcl-2 ↓
*Scutellaria baicalensis*	baicalein, luteolin, Oroxylin A, Baicalin dihydrobaicalein, wogonin	MMP-9 ↓, Hedgehog ↓, p38 ↑, MAPK ↑, Caspase ↑, ROS ↓
*Tripterygium wilfordii*	triptolide, tripdiolide, Celastrol, Wilforlide A	HIF-1α ↓, caspase ↑ PI3K/AKT/mTOR ↓, NF-κB ↓, MMP-2/MMP-9 ↓

Note: ↑ indicates promotion/activation/up-regulation; ↓ indicates inhibition/suppression/down-regulation. TNF-α: Tumor Necrosis Factor alpha; caspase-3/8/9: caspase-3/caspase-8/caspase-9 (cysteine-aspartic proteases); p53: Tumor protein p53; ROS: Reactive Oxygen Species; Bax: Bcl-2-associated X protein; Bcl-2: B-cell lymphoma-2; PI3K: Phosphoinositide 3-kinase; AKT: Protein kinase B; mTOR: Mechanistic Target of Rapamycin; MITF: Microphthalmia-associated transcription factor; E-cadherin: Epithelial cadherin; Nrf2: Nuclear factor erythroid 2-related factor 2; HO-1: Heme oxygenase-1; NF-κB: Nuclear Factor Kappa-light-chain-enhancer of activated B cells; AP-1: Activator protein-1; COX-2: Cyclooxygenase-2; miR-222-3p: MicroRNA 222-3p; Notch: Notch protein family; PD-L1: Programmed death-ligand 1; ERK: Extracellular signal-regulated kinase; TLR4: Toll-like receptor 4; MMPs: Matrix metalloproteinases; CD8+ T: CD8-positive T cells; CD4+ T: CD4-positive T cells; IL-6/IL-8: Interleukin-6/Interleukin-8; Cyclin D1/CDK4/6: Cyclin D1/Cyclin-dependent kinase 4/6; p21/p27: Cyclin-dependent kinase inhibitors p21 and p27; STAT1: Signal Transducer and Activator of Transcription 1; NQO1: NAD(P)H Quinone Dehydrogenase 1; HIF-1α: Hypoxia-inducible factor 1-alpha; GSK-3β: Glycogen synthase kinase-3 beta; EMT: Epithelial–Mesenchymal Transition; GSTM3: Glutathione S-transferase Mu 3; FNR: Ferredoxin-NADP+ reductase; MAPK: Mitogen-activated protein kinase; Hedgehog: Hedgehog signaling pathway; MyD88: Myeloid differentiation primary response gene 88; ER: Estrogen receptor; H2A.Z.1–E2F1: Histone variant H2A.Z.1–E2F transcription factor 1 axis; p38: p38 mitogen-activated protein kinase.

**Table 2:** Barrier–Solution–Evidence Matrix for Technology-Enabled Skin Cancer Therapies.

Barrier	Technology Solution	Example	Translational Relevance	Evidence Level
Poor skin penetration	Nanostructured lipid carriers	Quercetin and resveratrol gel	Enhances dermal deposition and bioavailability	in vivo, BALB/c nude mice
Tumor targeting	Targeted nanoparticles	Berberine-loaded nanoparticle	Improved tumor targeting and efficacy	in vivo, B16 melanoma xenograft model
Instability/Low bioavailability	Liposome–siRNA systems	Curcumin and STAT3 siRNA	Synergistic effect, enhanced apoptosis	in vitro, B16 cells
Co-delivery for synergy	Co-delivery platforms	Curcumin and siRNA	Enhanced tumor cell apoptosis, chemo-immunomodulation	in vivo, SKH-1 mice

**Table 3:** Evidence level, safety/metabolism concerns, and translational status of representative natural bioactive compounds and formulations.

Compound	Evidence and Model	Safety and Metabolism Concern	Translational Status
Triptolide (*Tripterygium wilfordii*)	Melanoma and cSCC; mainly in vitro and animal models; NF-κB, PI3K/AKT/mTOR, apoptosis, autophagy, DNA damage response	Narrow therapeutic window; hepatotoxicity, nephrotoxicity, and multi-organ toxicity; hepatic CYP450/P-gp-related exposure	No direct clinical trial for skin cancer treatment identified
Celastrol (*Tripterygium wilfordii*)	Melanoma models; mainly in vitro and animal models; ROS-mitochondrial apoptosis, PI3K/AKT/mTOR, HIF-1α	Poor water solubility, short half-life, low bioavailability, narrow therapeutic window, and systemic toxicity	Preclinical; nanoformulation and structural-modification strategies proposed
Curcumin (*Curcuma longa*)	UV-induced skin cancer, melanoma, and SCC-related models; NF-κB/AP-1, COX-2, Nrf2, PI3K/AKT/mTOR, Notch	Generally favorable safety profile, but limited by poor absorption, rapid metabolism, and low systemic exposure	Evaluated for radiation dermatitis; liposomal curcumin has entered cancer trials, but not directly for skin cancer treatment
Resveratrol (*Polygonum cuspidatum*)	NMSC/cSCC-related and photoprotection models; p53 induction, survivin suppression, ROS, NF-κB, MMPs	Generally well tolerated, but limited by rapid metabolism and low bioavailability	Topical gel registered for melasma; bioadhesive nanoparticle sunscreen trial related to photoprotection
Berberine (*Coptis chinensis*)	Melanoma models; PI3K/AKT/mTOR, EMT-related proteins, MITF/ERK/GSK3β	Low bioavailability; formulation-dependent delivery and systemic exposure need evaluation	Targeted nanoparticle delivery remains preclinical
Baicalein (*Scutellaria baicalensis*)	Melanoma and UV-damage models; NF-κB, MMP-2/9, PI3K/AKT/mTOR, Nrf2/HO-1	Pharmacokinetic validation and reproducibility across models remain insufficient	Preclinical
Tanshinone IIA and cryptotanshinone (*Salvia miltiorrhiza*)	Melanoma and UV-damage models; STAT1/PTGS2, AMPK/mTOR/HIF-1α, Nrf2	Solubility, exposure, and formulation stability need optimization	Preclinical formulation development
Natural-product-containing bioadhesive nanoparticle sunscreen	Human UVR-induced skin damage; phase 1 photoprotection-related study	Local irritation, sensitization, systemic absorption, and long-term safety require evaluation	ClinicalTrials.gov photoprotection-related study

## Data Availability

No new data were created or analyzed in this study. Data sharing is not applicable to this article.
